# Immunohistochemical expression of SPARC in odontogenic keratocysts: a comparative study with other odontogenic cysts

**DOI:** 10.1186/s12903-024-03978-4

**Published:** 2024-02-12

**Authors:** Sopee Poomsawat, Sirada Choakdeewanitthumrong, Sirima Sanguansin, Ounruean Meesakul, Theerachai Kosanwat

**Affiliations:** 1 Department of Oral and Maxillofacial Pathology, Faculty of Dentistry, Mahidol University, 6 Yothi Street, Ratchathewi, Bangkok, 10400 Thailand; 2 Department of Oral Biology, Faculty of Dentistry, Mahidol University, Bangkok, Thailand

**Keywords:** Calcifying odontogenic cyst, Dentigerous cyst, Odontogenic keratocyst, Radicular cyst, SPARC

## Abstract

**Background:**

Secreted protein acidic and rich in cysteine (SPARC) has been shown to modulate aggressive behavior in several benign and malignant tumors. Little is known about SPARC expression in odontogenic keratocyst (OKC), an odontogenic cyst with an aggressive nature. To the best of our knowledge, only one study has been investigated the expression of this protein in OKCs. This study aimed to characterize SPARC expression in OKCs. Additionally, to determine whether SPARC is associated with aggressive behavior in OKCs, SPARC expression in OKCs was compared with radicular cysts (RCs), dentigerous cysts (DCs) and calcifying odontogenic cysts (COCs). These odontogenic cysts showed no or less aggressive behavior.

**Methods:**

SPARC expression was evaluated in 38 OKCs, 39 RCs, 35 DCs and 14 COCs using immunohistochemistry. The percentages of positive cells and the intensities of immunostaining in the epithelial lining and the cystic wall were evaluated and scored.

**Results:**

Generally, OKCs showed similar staining patterns to RCs, DCs and COCs. In the epithelial lining, SPARC was not detected, except for ghost cells in all COCs. In the cystic wall, the majority of positive cells were fibroblasts. Compared between 4 groups of odontogenic cysts, SPARC expression in OKCs was significantly higher than those of RCs (*P* < 0.001), DCs (*P* < 0.001) and COCs (*P* = 0.001).

**Conclusions:**

A significant increase of SPARC expression in OKCs compared with RCs, DCs and COCs suggests that SPARC may play a role in the aggressive behavior of OKCs.

## Background

Secreted protein acidic and rich in cysteine (SPARC), also termed osteonectin, BM-40 and 43 K protein, is a Ca^2+^- binding glycoprotein in a family of matricellular proteins. It does not primarily serve as the structure of extracellular matrix (ECM), but mainly acts to modulate cell-cell and cell-matrix interactions by binding to structural proteins of ECM such as collagens, fibronectin and laminins [1]. SPARC also interacts with matrix metalloproteinases (MMPs) and many growth factors such as fibroblast growth factor-2 (FGF-2), vascular endothelial growth factor (VEGF) and transforming growth factor- β1 (TGF- β1). The interaction of SPARC and these various molecules modulates important cellular behaviors such as cell proliferation, differentiation, adhesion and migration [1, 2].

During tissue development and differentiation, SPARC expression is initially intense but it declines in most organs after maturation. Eventually, SPARC is restrictly expressed in post-developmental tissues with high ECM turnover such as bone and gut mucosa [3]. However, SPARC expression was upregulated during angiogenesis [3], inflammation [4], wound-healing [4] and tumor development [5].

In tumorigenesis, SPARC is expressed in tumor cells and surrounding stromal fibroblasts, sometimes designated as tumor-associated fibroblasts. This pattern of SPARC expression has been reported in several cancers such as lung, breast, colorectal and oral cancers [6–9]. Overexpression of epithelial and fibroblast SPARC was observed in oral squamous cell carcinoma (OSCC). This finding suggests that SPARC may play an important role during oral carcinogenesis. High SPARC expression in fibroblasts of OSCC may alter tumor microenvironment and thus affect tumor behavior [9]. Drev et al. conducted a migration assay and a 3D co-culture system in colorectal cancer cell lines. They found that SPARC-derived fibroblasts promoted migration velocity and depth of invasion of these cancer cells. These results imply that fibroblast SPARC may modulate aggressive behavior of colorectal cancer by enhancing cancer cell migration and invasion [8].

SPARC may also promote aggressiveness in ameloblastoma. Ameloblastoma is the most common benign odontogenic tumor. Despite its benign nature, ameloblastoma exhibits locally aggressive behavior [10]. Shen et al. investigated the expression of MMP-1, -2, -9 and SPARC in ameloblastoma and found that SPARC was co-expressed only with MMP-9. The interaction between SPARC and MMP-9 stimulates the proteolysis of ECM [11]. Additionally, angiogenesis is promoted by both SPARC and MMP-9. These mechanisms collectively contribute to the local aggressiveness of ameloblastoma [11]. In support to Shen’s study, Indirapriyadarsini et al. demonstrated a positive correlation between SPARC and MMP-9 expression in ameloblastoma [12]. It is of interest that SPARC may also be involved in aggressive behavior of odontogenic keratocysts (OKCs). OKCs are odontogenic cysts that cause large bony destruction and possess high recurrent rate [10]. It is well-accepted that OKCs show aggressive behavior. Moreover, some authors considered that OKCs should be classified as benign odontogenic tumors [13]. Due to their high recurrence, aggressive treatment approaches such as enucleation with peripheral ostectomy or with Carnoy’s solution, as well as surgical resection are recommended for managing OKCs [14]. Hong et al. studied SPARC expression in OKCs and found that SPARC was strongly expressed in fibroblasts of the cystic wall [15]. Until now, only one study of SPARC in OKCs has been conducted in English-language literature [15]. Therefore, the role of SPARC in OKCs remains largely unknown.

Due to the paucity of studies regarding SPARC expression in OKCs, this study aimed to evaluate SPARC expression in OKCs. Additionally, to determine whether SPARC is associated with aggressive behavior in OKCs, we compared SPARC expression in OKCs with other odontogenic cysts with no or less aggressive behavior including radicular cysts (RCs), dentigerous cysts (DCs) and calcifying odontogenic cysts (COCs). To the best of our knowledge, the expression of SPARC in RCs, DCs and COCs has never been studied. RCs are cysts of inflammatory origin. It is associated with the apical area of a non-vital tooth. Most RCs are treated by conventional endodontic treatment, but in large RCs, enucleation may be required [10]. DCs are considered to be cysts of developmental origin and are usually small and symptomless. This cyst is attached to the cervical area of an unerupted tooth and is commonly treated by total enucleation along with the impacted tooth removal [10]. Both RCs and DCs show excellent prognosis. Recurrence of RCs is extremely rare and that of DCs has never been reported [10]. COCs are rare odontogenic cysts characterized by the presence of ghost cells within epithelial lining [10]. The majority of COCs shows an indolent growth with asymptomatic swelling [16]. Enucleation is the treatment of choice for COCs and recurrence is rarely reported [10, 16].

## Methods

### Specimens

Four types of odontogenic cysts comprising 38 OKCs, 39 RCs, 35 DCs and 14 COCs were retrieved from departmental archives of Department of Oral and Maxillofacial Pathology, Mahidol University. Inclusion criteria for RCs required information from biopsy-requested records confirming the association of a cystic lesion with the apical area of a non-vital tooth. A radiographic feature indicating an unilocular radiolucency around the crown of an unerupted tooth was required for each DC. In OKC group, patients associated with nevoid basal cell carcinoma syndrome were excluded from our study. Histopathological diagnoses of all cases were confirmed by a board-certified oral pathologist (TK) using criteria according to WHO classification 2017 [10]. All RCs were lined by non-keratinized stratified squamous epithelium. Most DCs were lined by non-keratinized stratified squamous epithelium, although a few cases were lined by 2–3 cell layers thick cuboidal epithelium. The lining of OKCs were characterized by a uniform thickness of parakeratinized stratified squamous epithelium with a flat epithelial-connective tissue interface, basal cell palisading, and corrugated surface. For COCs, all cysts were lined by ameloblastoma-like epithelium with accumulations of ghost cells [10]. Only OKCs, DCs and COCs that presented no or minimal inflammation in histopathological examinations were selected. The present study was approved by the institutional ethics committee (COE.No.MU-DT/PY-IRB 2022/043.1609 ) and was conducted according to the guidelines of the Declaration of Helsinki.

### Immunohistochemistry

New 4-µm thickness sections were cut from the formalin-fixed paraffin-embedded blocks and mounted on glass slides coated with aminopropyltriethoxysilane (Sigma Chemical Co., St Louis, MO, USA). Sections were deparaffinized and rehydrated. Endogenous peroxidase activity was inhibited by incubating in 3% H_2_O_2_. Antigen retrieval was performed by heating the sections in a microwave for 15 min in 10 mM citrate buffer pH 6.0. After washing with 0.1% Tween 20 (MERCK-Schuchardt, Hohanbrunn, Germany) in phosphate-buffered saline (PBS), sections were treated with 5% bovine serum albumin (Sigma Chemical Co.) for 30 min to block non-specific antigens. Then the sections were treated with a primary antibody for two hours at room temperature. The primary antibody used in this study was against SPARC (Santa Cruz Biotechnology; SC-73,472) diluted at 1:200. The sections were subsequently incubated with peroxidase-conjugated secondary antibody (Dako Envision System, Dako Corporation, Glostrup, Denmark) for 30 min. After three washes of 0.1% Tween 20 in PBS, the immunoreaction was visualized by freshly made diaminobenzidine (Sigma Chemical Co.). Finally, the sections were counterstained with Mayer’s hematoxylin. Negative controls were accomplished by omitting the primary antibody and replacing with Tris buffered saline. For positive controls, sections of an oral squamous cell carcinoma known to express cytoplasmic SPARC were included in each run. All sections were processed under the same conditions.

### Evaluation of SPARC expression

The percentage of positive cells was semiquantitatively accessed by examining the entire tissue section using a 100x magnification. Each section was graded into one of the following groups: 0 (0–4%), 1 (5–24%), 2 (25–49%), 3 (50–74%), or 4 (75–100%). The intensity of immunostaining was graded as 0 (negative), 1 (light yellow), 2 (yellow brown), or 3 (dark brown). Subsequently, the score for each case was calculated by multiplying the percentage of positive cells by the staining intensity. This score was classified as follows: (+, low score) score 0–2, (+ +, intermediate score) score 3–5, (+ + +, high score) score 6–8, and (+ + + +, very high score) score 9–12. This scoring system was previously used to evaluate SPARC expression [11]. In each case, the score was independently investigated by two oral pathologists (SP and TK). Disagreement cases were discussed until consensus was reached.

### Statistical analysis

The nonparametric test was used in the current study. Data were analyzed using PASW Statistics for Windows, Version 18.0. (SPSS Inc., Chicago, USA). The scores of SPARC expression between the four groups of odontogenic cysts were compared using the Kruskal–Wallis test and post hoc Dunn’s multiple comparison test. A value of *P* < 0.05 was considered statistically significant.

## Results

### Patterns of SPARC expression in OKCs, RCs, DCs and COCs

**Fig. 1:**
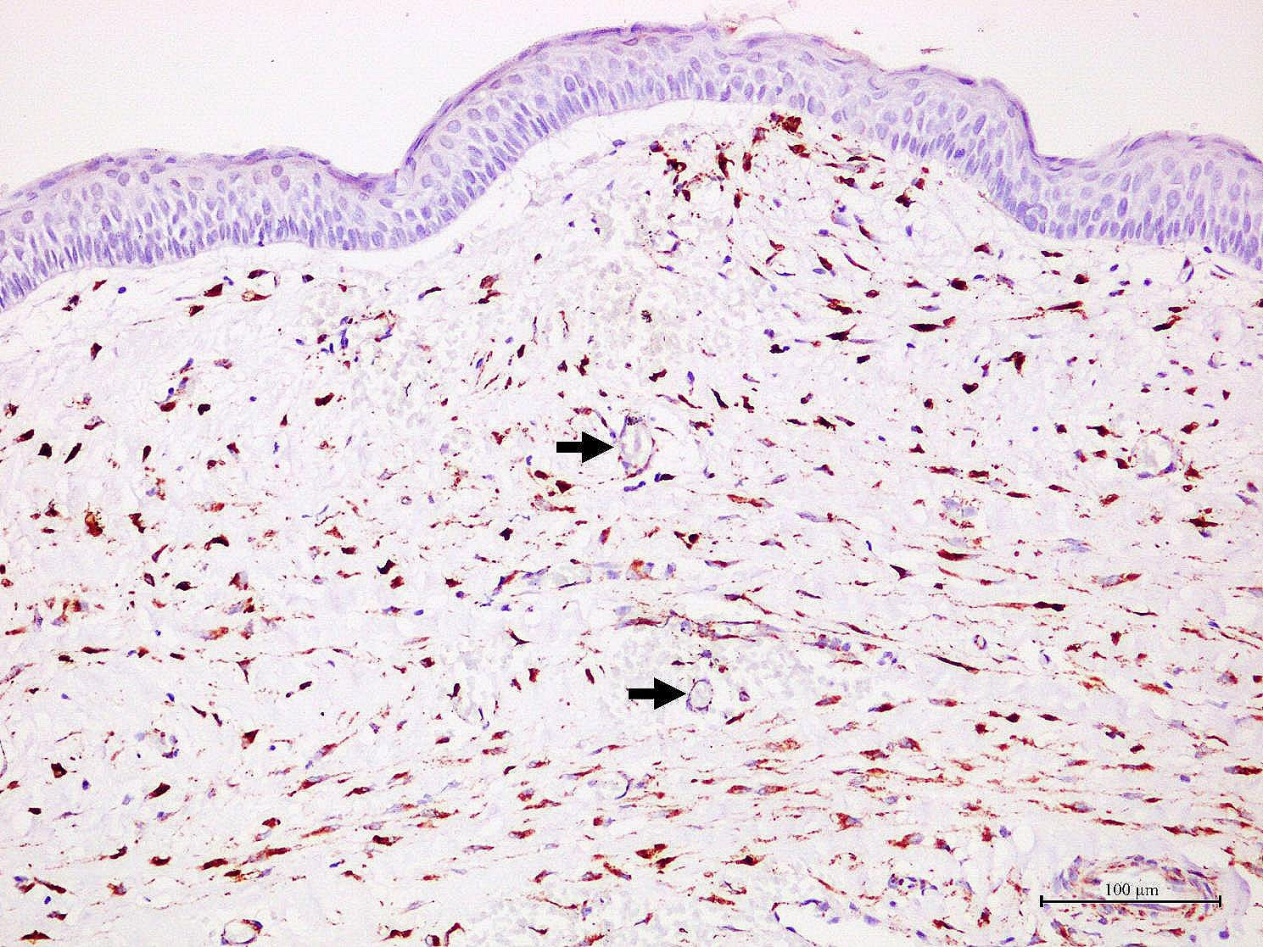
In a representative of odontogenic keratocyst, dark brown staining of SPARC is found in the cytoplasm of numerous fibroblasts. Note that the endothelial cells lining some blood vessels (arrows) are also positive to SPARC.

**Fig. 2:**
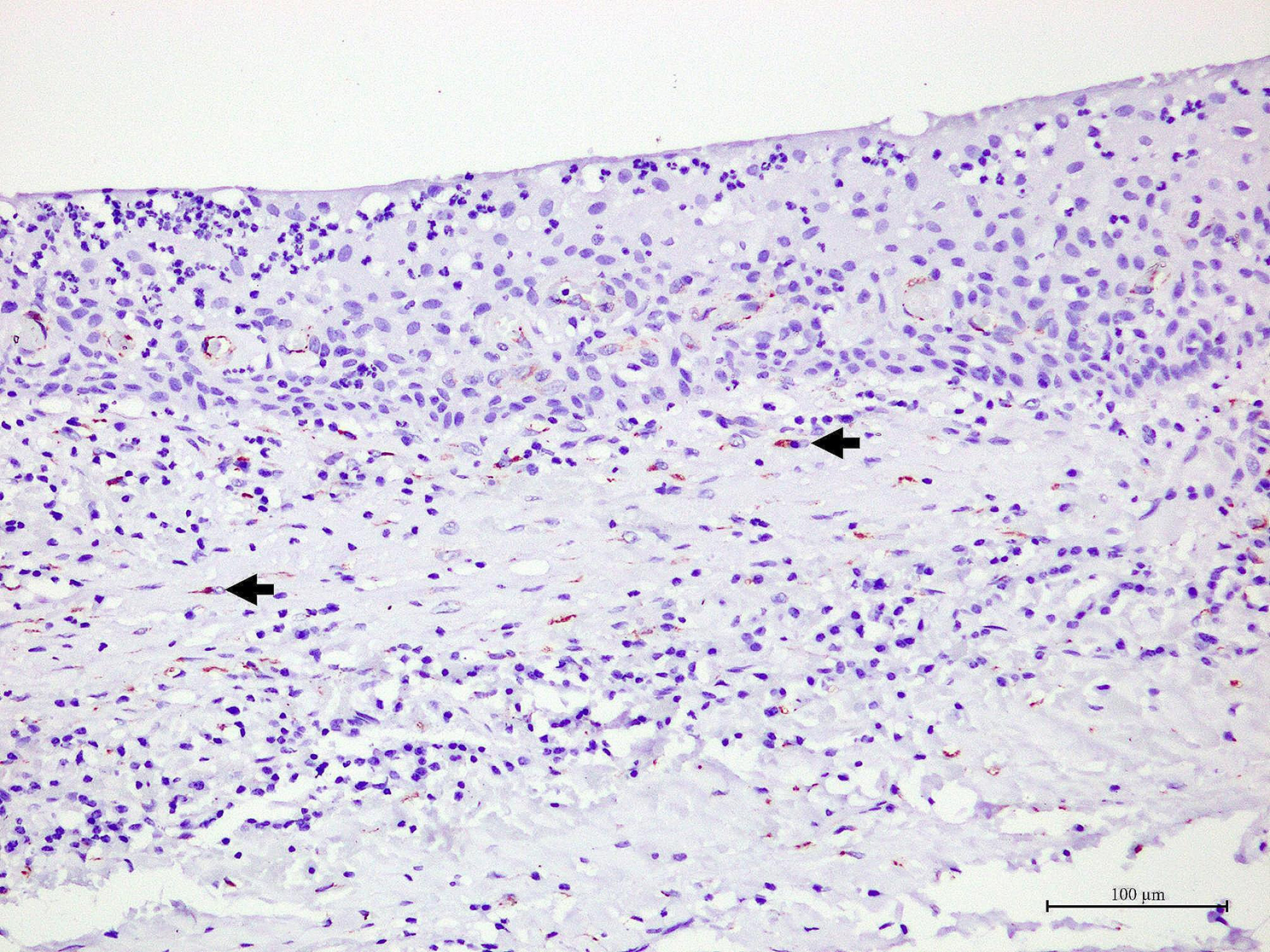
In a representative of radicular cyst, yellow brown staining of SPARC is found in the cytoplasm of a small number of fibroblasts (arrows)

**Fig. 3:**
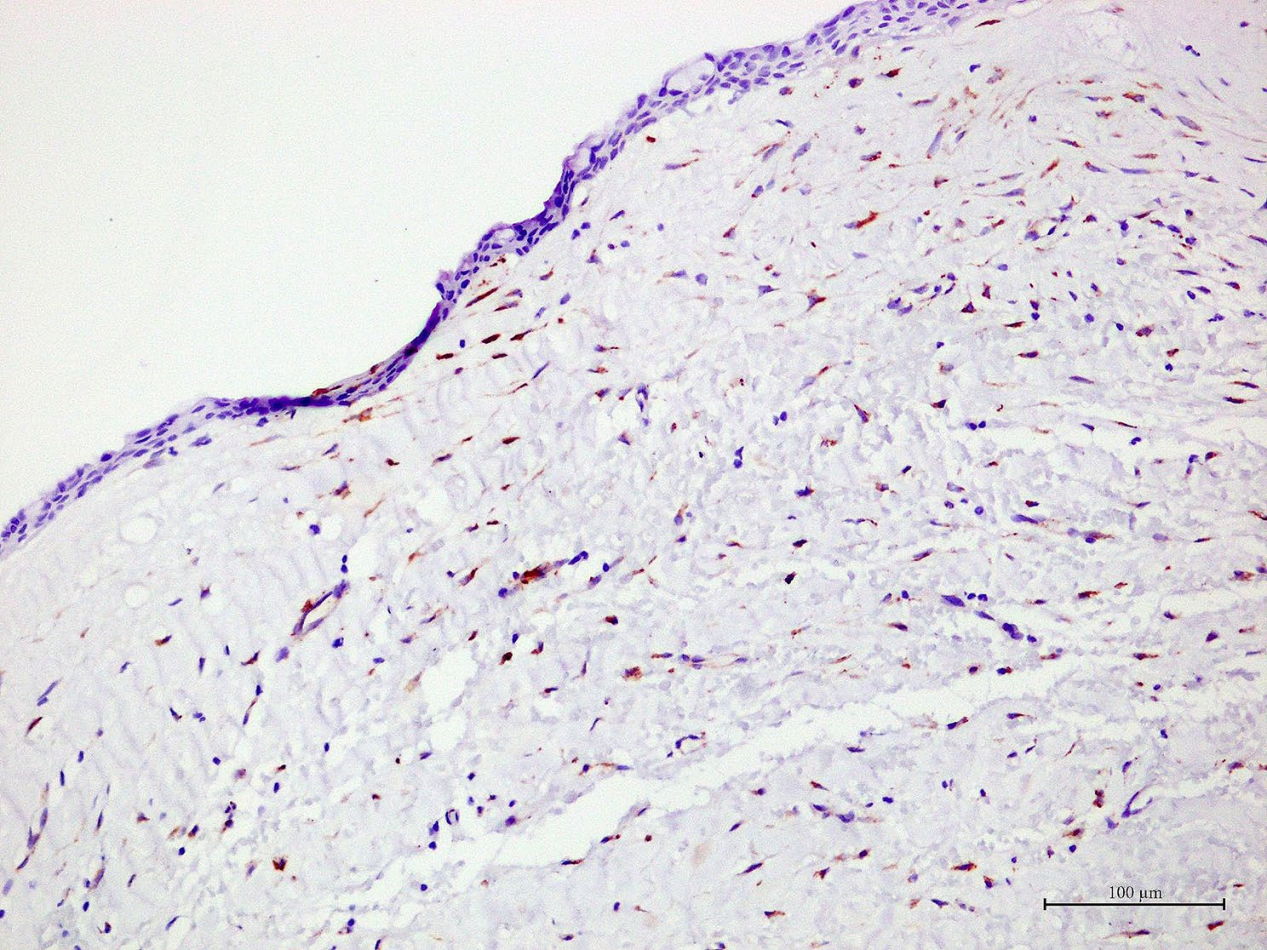
In a representative of dentigerous cyst, yellow brown staining of SPARC is observed in the cytoplasm of several fibroblasts

**Fig. 4:**
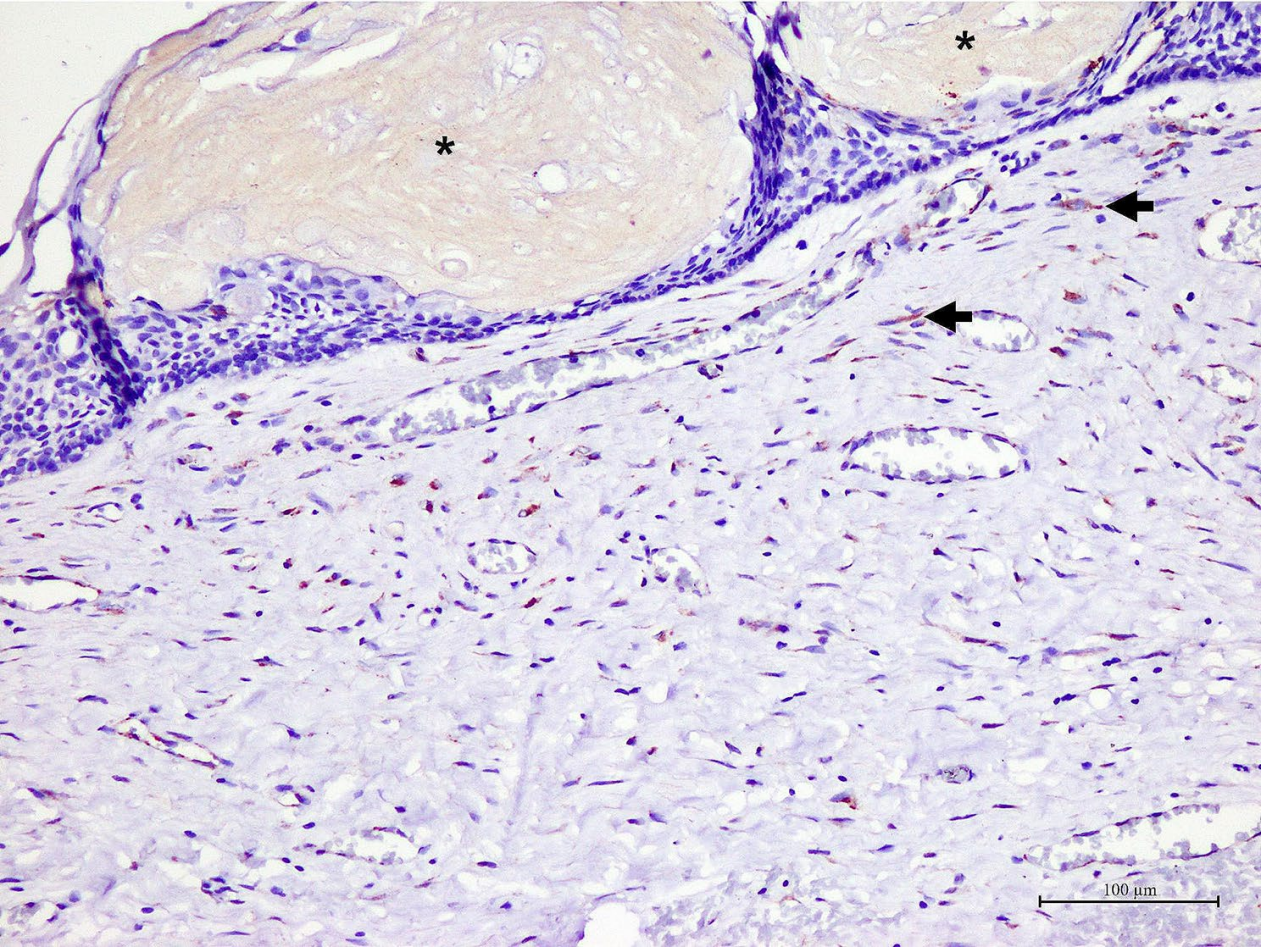
In a representative of calcifying odontogenic cyst, yellow brown staining of SPARC is found in the cytoplasm of few fibroblasts (arrows). Note that SPARC also decorates ghost cells (asterisks)

Generally, OKCs showed similar staining patterns to RCs, DCs and COCs. SPARC positive cells were predominantly found in the fibrous wall, particularly the fibroblasts. Various intensities, ranging from light yellow to dark brown, were observed in fibroblast cytoplasm. The majority of these fibroblast-positive cells were large, spindle or stellate-shaped cells. Additionally, they were often found in the fibrous wall close to the lining epithelium. In addition to fibroblasts, the endothelial cells lining some blood vessels, nerves and osteoblasts in bone shells were positive to SPARC. Representative samples of SPARC in odontogenic cysts are shown in Figs. 1, 2, 3 and 4. In the epithelial lining, SPARC was not detected except for ghost cells in all COCs (Fig. 4 ).

### Fibroblast score of SPARC in OKCs, RCs, DCs and COCs

Because the majority of positive cells were fibroblasts, the scoring system as described in the material and methods section was applied to evaluate fibroblast-positive cells. Fibroblast scores of SPARC in all odontogenic cysts are summarized in Table 1. The majority of OKCs (21/38 cases, 55.3%) were graded as very high score (+ + + +). In contrast to OKCs, most RCs (28/39 cases, 71.8%), DCs (17/35 cases, 48.6%), and COCs (9/14 cases, 64.3%) were graded as low score (+). OKCs had significantly higher fibroblast scores than RCs (*P* < 0.001), DCs (*P* < 0.001) and COCs (*P* = 0.001). No significant difference of fibroblast scores between the remaining odontogenic cysts was observed.

**Table 1:** Fibroblast scores of SPARC in odontogenic cysts

Types of cysts (Total number of cases)	Number of cases				*P* value
	+	+ +	+ + +	+ + + +	
Odontogenic keratocysts (38)	6	2	9	21	
Radicular cysts (39)	28	3	6	2	< 0.001*
Dentigerous cysts (35)	17	10	6	2	< 0.001*
Calcifying odontogenic cysts (14)	9	2	2	1	0.001*

* Statistically significant compared with odontogenic keratocysts

+ Low score (score 0–2); + + Intermediate score (score 3–5); + + + High score (score 6–8); + + + + Very high score (score 9–12)

## Discussion

This study evaluated SPARC expression in OKCs and compared with those of RCs, DCs and COCs. SPARC was observed in all four cyst types. In general, OKCs showed similar staining patterns to RCs, DCs and COCs. SPARC was predominantly localized in the cytoplasm of fibroblasts within the cystic wall. Epithelial lining cells were not decorated by SPARC, except for ghost cells in COCs. Compared among the four groups of odontogenic cysts, SPARC expression in OKCs was significantly higher than those of RCs, DCs and COCs.

The expression of SPARC in RCs and DCs has never been reported in English-language literature. Our study showed that SPARC was found in the fibroblasts in the cystic wall of RCs and DCs, but not in the epithelial lining. Generally, expression of SPARC was low in RCs and DCs except for a few cases.

To date, no study has investigated SPARC expression in COCs. Unlike other odontogenic cysts, COCs showed SPARC expression not only in fibroblasts of the cystic wall, but also cells in the epithelial lining. In this study, SPARC was found in ghost cells of all examined COCs. Ghost cells have been shown to be accumulated enamel-related proteins [17] and matrix glycoproteins [18]. A prior study has demonstrated that ghost cells are immunoreactive to several extracellular matrix including laminins 1 and 5, collagen type IV and fibronectin [18]. As SPARC is one of the matricellular proteins, our finding of SPARC expression in ghost cells is not surprising. It has been shown that SPARC, also known as osteonectin, stimulates the calcification process of bone. This occurs because SPARC can bind to collagens and release calcium ions [19]. Because ghost cells in COCs frequently undergo dystrophic calcification [10], SPARC may be involved in the calcification process in ghost cells.

The expression of SPARC in OKCs in our study is consistent with that of Hong’s study. In their study, SPARC was found in the cytoplasm of fibroblasts but not in the epithelial lining [15]. Comparing among the four groups of odontogenic cysts, we found that OKCs showed significantly higher fibroblast SPARC expression than those of RCs, DCs and COCs. These results suggest that SPARC may be involved in the aggressive behavior of OKCs. This is because OKCs have an aggressive nature while RCs, DCs and COCs show no or less aggressive behavior [10, 16]. In support to our suggestion, increased SPARC levels have been shown to increase the production and activity of MMPs, leading to matrix degradation in breast [20] and prostate cancers [21]. In ameloblastoma, SPARC expression showed a significant correlation with MMP-9 expression, suggesting that SPARC participates in local aggressiveness of this tumor [11, 12]. Because MMP-9 can lead to bone resorption by cleaving various components of bony ECM such as collagens type IV and V, proteoglycans, and elastin [22]. Moreover, MMP-9 also degraded collagen type I [23], a major component of bone ECM, leading to alteration of bone ECM organization and reduction of bone strength [24]. These aforementioned mechanisms may cause a significant bone resorption in OKCs and contribute to their aggressiveness.

The high expression of SPARC in OKCs may have potential implications for alternative treatments of OKCs. Due to its aggressive nature characterized by large bone destruction and high recurrence rate, a surgical resection was recommended for approaching OKCs with large or multiple recurrences. Although this technique showed a good outcome, it also resulted in significant morbidity [14]. SPARC has been shown to be an important factor for drug accumulation [25]. Because SPARC has a high affinity with nanoparticle albumin-bound (NAB) molecule, lesions with high SPARC production show high response to drugs using NAB delivery technology [26, 27]. In patients with head and neck cancer, NAB-paclitaxel drug remarkably reduced tumor size in SPARC-positive patients compared with that of the SPARC-negative group [27]. Based on these previous works, this drug technology may provide an alternative treatment for OKCs.

## Conclusions

SPARC expression was predominantly observed in fibroblasts of OKCs, RCs, DCs and COCs. A significant increase in fibroblast SPARC was found in OKCs compared with RCs, DCs and COCs, suggesting that SPARC may play a role in the aggressive behavior of OKCs.

## Data Availability

The datasets used and analyzed during the current study available from the corresponding author on reasonable request.

## Abbreviations

COC: Calcifying odontogenic cyst
DC: Dentigerous cyst
ECM: Extracellular matrix
FGF-2: Fibroblast growth factor-2
MMP: Matrix metalloproteinase
NAB: Nanoparticle albumin-bound
OKC: Odontogenic keratocyst
OSCC: Oral squamous cell carcinoma
RC: Radicular cyst
SPARC: Secreted protein acidic and rich in cysteine
TGF- β1: Transforming growth factor- β1
VEGF: Vascular endothelial growth factor
